# Fluid sparing and norepinephrine use in a rat model of resuscitated haemorrhagic shock: end-organ impact

**DOI:** 10.1186/s40635-018-0212-3

**Published:** 2018-11-12

**Authors:** Sophie Dunberry-Poissant, Kim Gilbert, Caroline Bouchard, Frédérique Baril, Anne-Marie Cardinal, Sydnée L’Ecuyer, Mathieu Hylands, François Lamontagne, Guy Rousseau, Emmanuel Charbonney

**Affiliations:** 1 Département de Médecine, Université de Montréal, C.P. 6128 Succursale Centre-ville, Montréal, QC H3C 3J7 Canada; 2 Centre de Recherche Hôpital du Sacré-Cœur de Montréal (HSCM), 5400 boul. Gouin Ouest, Montréal, QC H4J 1C5 Canada; 3 Université de Montréal, 2900 Edouard Montpetit Blvd, Montréal, QC H3T 1J4 Canada; 4 Département de chirurgie, Université de Sherbrooke, 3001- 12e avenue Nord, Sherbrooke, QC J1H 5N4 Canada; 5 Centre de recherche du CHU de Sherbrooke, 3001- 12e avenue Nord, Sherbrooke, QC J1H 5N4 Canada; 6 Department of Medicine, Université de Sherbrooke, 3001- 12e avenue Nord, Sherbrooke, QC J1H 5N4 Canada; 7 Département de pharmacologie et physiologie, Université de Montréal, C.P. 6128 Succursale Centre-ville, Montréal, QC H3C 3J7 Canada

**Keywords:** Haemorrhage shock, Vasopressors, Norepinephrine, Fluid sparing, End-organ impact

## Abstract

**Background:**

Haemostasis and correction of hypovolemia are the pillars of early haemorrhage shock (HS) management. Vasopressors, which are not recommended as first-line therapy, are an alternative to aggressive fluid resuscitation, but data informing the risks and benefits of vasopressor therapy as fluid-sparing strategy is lacking. We aimed to study its impact on end organs, in the setting of a haemodynamic response to the initial volume resuscitation.

**Methods:**

Following controlled HS (60 min) induced by blood withdrawal, under anaesthesia and ventilation, male *Wistar* rats (*N* = 10 per group) were randomly assigned to (1) sham, (2) HS with fluid resuscitation only [FR] and (3) HS with fluid resuscitation to restore haemodynamic (MAP: mean arterial pressure) then norepinephrine [FR+NE]. After a reperfusion time (60 min) during which MAP was maintained with fluid or norepinephrine, equipment was removed and animals were observed for 24 h (*N* = 5) or 72 h (*N* = 5) before euthanasia. Besides haemodynamic parameters, physiological markers (creatinine, lactate, pH, PaO_2_) and one potential contributor to vasoplegia (xanthine oxidase activity) were measured. Apoptosis induction (caspase 3), tissue neutrophil infiltration (MPO: myeloperoxidase) and illustrative protein markers were measured in the lung (Claudin-4), kidney (KIM-1) and brain amygdala (Iba1).

**Results:**

No difference was present in MAP levels during HS or reperfusion between the two resuscitation strategies. FR required significantly more fluid than FR+NE (183% vs 106% of bleed-out volume; *p* = 0.003), when plasma lactate increased similarly. Xanthine oxidase was equally activated in both HS groups. After FR+NE, creatinine peaked higher but was similar in all groups at later time points. FR+NE enhanced MPO in the lung, when Claudin-4 increased significantly after FR. In the brain amygdala, FR provoked more caspase 3 activity, MPO and microglial activation (Iba1 expression).

**Conclusion:**

Organ resuscitation after controlled HS can be assured with lesser fluid administration followed by vasopressors administration, without signs of dysoxia or worse evolution. Limiting fluid administration could benefit the brain and seems not to have a negative impact on the lung or kidney.

## Background

Polytrauma is a major cause of mortality worldwide [1], and haemorrhagic shock (HS) is the main pathway to early mortality after trauma [2].

According to current guidelines, initial management of HS consists of rapid haemostasis with concurrent repletion of blood volume with warmed crystalloid and blood products. Fluid resuscitation restores circulating volume in order to compensate for decreased end-organ perfusion. However, large volumes of intravenous fluids can lead to dilutional coagulopathy, hypothermia and acidosis [3, 4], as well as tissue oedema and prolonged mechanical ventilation [5].

The debate surrounding the optimal resuscitation strategy remains unresolved, both in the pre-hospital setting and during late resuscitation [5, 6]. Vasopressors, which are not currently recommended for HS, constitute a potential fluid-sparing strategy [7]. They are commonly used when polytrauma is associated with traumatic brain injury, presumably to avoid low cerebral perfusion pressures [8, 9]. However, existing clinical data remains inconclusive and their net effects remain uncertain [10].

Vasopressors’ ultimate impact on tissue perfusion is unclear, as any benefits of higher arterial pressure may be offset by increased microvascular resistance. Microcirculatory derangements and vasoplegia commonly persist despite adequate fluid resuscitation in the setting of haemorrhagic shock [11]. Organ failure can then result, partly through the release of nitric oxide and peroxynitrites [12, 13]; interestingly, a large number of preclinical haemorrhagic shock models have studied the role of xanthine oxidase activation as a culprit [14, 15].

When used alone or in high doses in animal HS models, vasopressors are associated with increased mortality [16]; combined with fluid resuscitation, survival is improved compared to fluid resuscitation alone [16–18]. In another study, a restrictive fluid strategy combined with vasopressor therapy leads to better lung compliance and reduced pulmonary oedema but higher serum lactate concentrations [19].

The aim of our study was to compare the end-organ impact (lung, kidney and brain) of a mean arterial pressure (MAP)-directed fluid resuscitation with or without norepinephrine (NE), in a rat model of controlled HS. We hypothesized that the addition of NE would lower fluid requirements, which could have a differential biologic impact on different end organs. Besides haemodynamic, fluid-related and physiological variables, we also measured plasma levels of a vasoplegia contributor (xanthine oxidase), tissue apoptosis induction program (caspase 3) and tissue neutrophil infiltration (myeloperoxidase). Specific protein’s expression was chosen from the literature to illustrate phenomenon that could respond to differential resuscitation in the organs studied: for the kidney, the kidney injury molecule-1(KIM-1) was chosen as marker of the proximal tubular epithelium ischemia [20]; for the lung, Claudin-4 (a junctional protein) was chosen for its induced expression after lung injury and its role in fluid clearance [21]; and for the brain, the ionized calcium binding adaptor molecule 1 (Iba1) was chosen as an ischemia-sensitive protein, specifically expressed in microglia and reflecting inflammatory activation [22].

## Methods

### Experimental design

Thirty-three male Wistar rats weighing 400 to 450 g (aged 12 to 15 weeks; Charles River Canada, St-Constant, QC, Canada) were housed individually, under constant conditions (temperature 21–22 °C and humidity 40–50%), including a 12-h dark-light cycle beginning at 8 AM. Chow pellets and tap water were available ad libitum throughout the study. An acclimatization period of 3 days after delivery by the supplier was imposed before the rats were allocated to the experimental groups. We chose to use norepinephrine (NE), since it is widely available and used by clinicians [23]. The animals (*N* = 10) were randomly assigned to one of the three experimental groups: (1) sham, (2) HS with fluid resuscitation only [FR] and (3) HS with both fluid resuscitation and NE [FR+NE]. The number of animals per groups was based on the survival rate reported with similar doses of NE in a rat model [16]. On the morning of the experiments, two rats were prepared and anesthetized, then allocated in parallel to two of the experimental groups.

### Surgical procedure and haemorrhagic shock model (Fig 1)

Anaesthesia was induced by intraperitoneal ketamine/xylazine injection (60 and 10 mg/kg, respectively). Subsequently, the rats were intubated, ventilated under volume control parameters (6 ml/kg) and received oxygen (FiO2 100%). Anaesthesia was maintained with isoflurane (1–2%). After lidocaine administration (5 mg/kg SC), catheters were placed in the femoral vein (for blood subtraction, plasma sampling and fluid administration) and in the femoral artery (for monitoring of arterial pressure) under sterile conditions. Catheters were fixed with silk sutures. Oxygen saturation (pulse oximetry) and temperature (with a rectal probe) were monitored. Blood was drawn until the mean arterial pressure (MAP) fell to 30 mmHg [24], and the subtracted volume was recorded. This degree of hypotension was maintained for 60 min to reflect the clinical effects of trauma-induced hypovolemia and hypoperfusion. The sham group was equipped with catheters and observed for the same duration but blood was not removed.

**Fig. 1:**
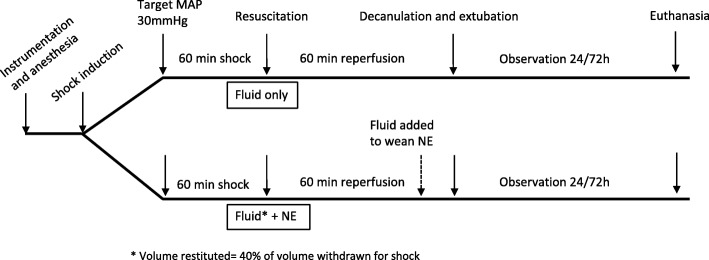
Experimental timeline*.* Surgical procedure and haemorrhagic shock model overview

In both groups, resuscitation was initiated with shed blood (stored at 37 °C with citrate) mixed with Ringer’s lactate (1:1). In the FR group, boluses of 1–2 ml were constantly administered, in order to maintain a MAP 55–60 mmHg. In the FR+NE group, rats were initially resuscitated with a bolus totalizing 40% of the blood volume initially withdrawn; this percentage had been evaluated in pilot experiments and determined as the minimal volume to reach the target MAP. Thereafter, an infusion of NE (0.1 mg\ml) was started at 2 mcg/100 g/h and titrated to target a MAP 55–60 mmHg for 45 min. Volume was then administered to wean NE and reach a MAP of 60 mmHg at anaesthesia interruption. After 60 min of reperfusion, catheters were removed, vessels ligated, and a subcutaneous analgesic injection (0.05 mg/kg buprenorphine) was administered. The rats were then extubated and allowed to eat and drink (water consumption was monitored) before their euthanasia at two pre-specified time points (24 h or 72 h; *N* = 5 per time point). Another buprenorphine injection was administered the next day. Three rats died in the first hour following reperfusion (< 10% of the total cohort), one in the FR+NE group and two in the FR group. Animals were added until five animals completed the experiment (24 and 72 h) in each group (FR, FR+NE). All animals that survived the initial resuscitation period survived until the final euthanasia.

As a measure of general recovering state and given the difference in fluid administration between the groups, weight loss till euthanasia and water intake during the first 24 h after HS were measured.

The animals were euthanized by decapitation under deep anaesthesia with intraperitoneal ketamine/xylazine injection (60 and 10 mg/kg, respectively). After euthanasia, organs (kidney, lung and brain) and plasma were retrieved, frozen in liquid nitrogen and stored at − 80 °C until analysis.

### Laboratory assays

Blood (0.5 ml) was collected at baseline, at the end of the reperfusion period (preceding decanulation) and at the time of euthanasia (24 h, 72 h). In all groups, creatinine level and arterial blood gases (pH, PaO_2_, lactate) were assessed at baseline, at reperfusion and at euthanasia (results pooled for 24 h and 72 h), using a VetScan i-Stat© (Abaxis Inc., California, USA)*.* Plasma xanthine oxidase activity was measured at reperfusion and at euthanasia, according to the protocol provided by the manufacturer (Xanthine Oxidase Fluorometric Assay Kit, Cayman Chemicals number 10010895).

Caspase 3 activity was measured according to a previously described protocol [25]. Tissues were homogenized by sonication in a lysis buffer and incubated for 30 min on ice. After three freeze/thaw cycles, tissue homogenates were centrifuged at 4 °C for 10 min. Enzymatic reactions were undertaken in a buffer with 25 mg of protein (confirmed by the Bradford method) and 40 μM of fluorescent substrates (Ac-DEVD-AMC and Ac-IETD-AMC for caspase 3). The reactions were assessed after a 3-h incubation in the dark at 37 °C and stopped with the addition of 0.4 M NaOH and 0.4 M glycine buffer. Fluorescence was quantified by spectrofluorometry (Photon Technology International, Lawrenceville, NJ, USA) at an excitation wavelength of 365 nm and an emission wavelength of 465 nm for caspase 3 activities.

Neutrophil accumulation in the tissue was estimated by myeloperoxidase assay as described previously [26]. Organ’s tissues were first weighed, added to a hexadecytrimethylamonium bromide (HTAB) buffer (0.5% HTAB + 50 mM potassium phosphate, pH 6.0) and pulverized by sonication. The lysates were subjected to three freeze/thaw cycles and centrifuged at 21,000*g* for 15 min. To quantify myeloperoxidase activity, 0.1 ml of supernatant was added to 2.9 ml of 50 mM sodium phosphate (pH 6.0) with 0.167 mg/ml of o-dianisidine hydrochloride and 0.0005% hydrogen peroxide. Absorbance of the orange product was then measured by spectrophotometer every 10 s at 460 nm for 2 min. Difference was generated between the maximum and minimum divided by 2 to estimate neutrophil accumulation.

### Western blotting

The increase in expression of selected proteins known to respond to systemic injury was measured in the end organ as a read-out of the HS-related ischemic damage. Briefly, tissues were homogenized by sonication in a lysis buffer (1% Triton X-100, 0.32 mol/l sucrose, 10 mmol/l Tris (pH 8.0), 5 mmol/l EDTA, 2 mmol/l DTT, 1 mmol/l PMSF, 10 mg/ml Leupeptin, 10 mg/ml Pepstatin A, 10 mg/ml Aprotinin). The tissue homogenates were incubated for 30 min at 4 °C and centrifuged at 10,000*g* for 15 min. Protein concentrations of the supernatant were quantified by the Lowry method. Aliquots of 100 mcg protein were loaded in polyacrylamide gels (10–15%) and migrated at 150 V for 75 min in a mini-gel apparatus (BioRad Laboratories, Hercules, CA, USA). Proteins were transferred to nitrocellulose membranes with a Trans-Blot semi-dry transfer cell (BioRad Laboratories). Using SNAP i.d. 2.0 system (Millipore, Etobicoke, Ontario, Canada), non-specific sites were blocked for 20 min, incubated in Odyssey blocking buffer (Li-CoR, Lincoln, Nebraska, USA) (1:1 with PBS). After PBS washing, membranes were incubated for 15 min with primary antibody 1:1000 (Novus biologicals NBP2-46655 pAb anti-TIM-1/KIM-1/HAVCR anti-rabbit: KIM-1), (rabbit pAb Claudin-4 abcam ab53156), (Rabbit Iba1 pAb Wako 019–19,741) and actin (anti-actin rabbit Ab sigma A2066). After washing, the membranes were incubated for 10 min with secondary antibody 1: 15,000 (anti-rabbit IRDye 800CW Li-Cor). The membranes were scanned with Odyssey LI-COR Clx, and band intensities were analysed with image studio. The same membranes were placed in stripping buffer (0.1 mol/l glycine, 1% SDS, pH 2.0, 1 h at room temperature) and reused with the same technique for ratio determination KIM-1/actin, Claudin-4/actin and Iba1/actin ratio.

### Statistical analysis

The data are reported as means (± SEM). Statistical analyses were performed by SPSS 19. Data were compared with analysis of variance (ANOVA), followed by Bonferroni correction for univariate multiple comparisons, when parametric. If variances were heterogeneous, Brown–Forsythe correction was followed by Games–Howell comparisons when applicable. Comparison of within groups variable changes were done using Kruskal–Wallis test. All *p* values compare either the HS groups to the sham group or the HS groups to one another. *p* < 0.05 was considered significant.

## Results

### Resuscitation, haemodynamic and monitoring parameters

At baseline, MAP and heart rate (HR) were comparable in all groups. Resuscitation and haemodynamic parameters during HS are displayed in Table 1. The bleed-out volumes of the two HS groups (FR vs FR+NE) were equivalent (15.6 ± 1.2 vs 17.1 ± 1.2 ml; *p* = 0.38). During HS, the lowest MAP reached was the same (27 ± 1 vs 26.5 ± 0.5 mmHg; *p* = 0.66), as well as the time spent under MAP of 30 mmHg (11.1 ± 1.6 vs 10.5 ± 1.1 min; *p* = 0.73). The FR+NE group was resuscitated with the pre-established limited volume (11 ± 1 ml), with a MAP ≥ 55 mmHg in all animals. Thereafter, when MAP fell below, NE was infused (total average dose of 9.2 mcg) until the end of the reperfusion period (60 min); additional fluid was then necessary (9.3 ± 1.7 ml), in order to wean NE, before extubation. The FR group received a total amount of 27.5 ± 1.5 ml of fluid for resuscitation and MAP maintenance. During the reperfusion period till extubation, the FR vs FR+NE lowest MAP reached was not different (54 ± 2 vs 51 ± 2 mmHg; *p* = 0.24), as well as the time spent under MAP of 55 mmHg (7.8 ± 1.6 vs 8.0 ± 1.1 min; *p* = 0.92).

**Table 1:** Resuscitation, haemodynamic and monitoring parameters

	Sham *N* = 10	HS: FR+NE *N* = 10	HS: FR *N* = 10
Bleed-out volume, ml	0	17.1 ± 1.2	15.6 ± 1.2
Resuscitation volume (%)	0	106 ± 11**	183 ± 17
Cumulative NE, mcg	0	9.2 ± 0.8	0
MAP, mmHg			
Baseline	65 ± 3	66 ± 2	65 ± 2
Shock	63 ± 2	29 ± 1*	29 ± 1*
Reperfusion	67 ± 5	59 ± 3	54 ± 2
HR, bpm			
Baseline	267 ± 13	263 ± 11	265 ± 18
Shock	266 ± 14	247 ± 11	248 ± 15
Reperfusion	273 ± 15	321 ± 11	394 ± 14
Temperature, ^°^C			
Baseline	35 ± 0.9	36 ± 0.2	34 ± 0.6
Shock	35 ± 0.8	36 ± 0.2	34 ± 0.6
Reperfusion	35 ± 0.6	37 ± 0.2	35 ± 0.4
Saturation, %			
Baseline	98 ± 0.5	98 ± 0.5	96 ± 0.7
Shock	98 ± 0.5	98 ± 0.5	96 ± 0.9
Reperfusion	97 ± 0.5	98 ± 0.5	97± 0.5

Data represented are those of animals who survived till their euthanasia. Values are reported as Mean ± SEM, during each defined period. FR = fluid resuscitation, FR+NE = fluid with norepinephrine, MAP = mean arterial pressure, HR = heart rate, bpm = beats per minute

*p < 0.001 vs shams; **FR+NE vs FR (*p* = 0.003)

At the time of extubation and decanulation, the group of rats resuscitated with fluids only (FR) had required more fluids than animals resuscitated with FR+NE (183% vs 106% of bleed-out volume; *p* = 0.003). During reperfusion, both HS groups were comparable and had lower MAP and higher HR than the sham group. No differences were noted in terms of saturation or temperature between groups (Table 1).

### Organ function and dysoxia

HS induced a significant rise in lactate at the end of reperfusion compared to baseline in the HS groups (*p* = 0.006), but serum lactate concentrations were comparable in both HS groups (Table 2). No difference was noted in terms of PaO_2_ or pH. Creatinine level increased significantly within HS groups (*p* = 0.037). At the end of reperfusion, creatinine was significantly increased compared to shams in the FR+NE group (absolute level and delta; *p* < 0.05). Delta creatinine was non-significantly higher in the group of FR+NE compared to FR after reperfusion (Delta A). When animals were euthanized, creatinine level (Delta B) in the FR vs FR+NE groups was not different, and similarly, no differences were noted between 24 h and 72 h creatinine levels in each arm. Animals lost more weight during the first 24 h and recovered partially at 72 h, with no difference between groups. Fluid intake was equal within the first 24 h for sham and HS groups (Table 3).

**Table 2:** Physiological parameters measured

	Sham *N* = 10	HS: FR + NE *N* = 10	HS: FR *N* = 10	P FR vs F+NE
Lactates, mmol/l				
Baseline	0.56 ± 0.11	0.66 ± 0.12	0.47 ± 0.07	NS
Reperfusion^#^	0.77 ± 0.20	1.50 ± 0.22	1.16 ± 0.27	NS
Delta lactate	0.21 ± 0.18	0.76 ± 0.34^**§**^	0.69 ± 0.29^**§**^	NS
pH				
Baseline	7.38 ± 0.03	7.42 ±0.03	7.48 ±0.04	NS
Reperfusion^#^	7.37 ± 0.01	7.37 ±0.03	7.41 ±0.03	NS
PaO2, mmHg				
Baseline	328 ± 31	336 ± 30	309 ± 49	NS
Reperfusion^#^	334 ± 28	339 ± 24	301 ± 18	NS
Creatinine, mg/dl				
Baseline	0.32 ± 0.05	0.33 ±0.03	0.29 ± 0.05	NS
Reperfusion^#^	0.34 ± 0.07	0.71 ±0.11^**§**^	0.53 ± 0.10	NS
Euthanasia	0.26 ± 0.08	0.32 ±0.06	0.26 ± 0.03	NS
Delta creatinine A^*^, mg/dl	0 ± 0.04	0.30 ±0.08^**§**^	0.20 ± 0.05	NS
Delta creatinine B^**^, mg/dl	0 ± 0.06	0 *±* 0.06	0 ± 0.06	NS

Values are reported as Mean ± SEM. ^#^Measured at the end of the reperfusion period

*Delta creatinine A (end of reperfusion-baseline); ** Delta creatinine B (at euthanasia-baseline). ^**§**^*p* < 0.05 vs sham

**Table 3:** Animal’s follow-up

	Sham	FR+NE	FR
Weight change, g			
24 h	− 24 ± 4	−19 ± 5	− 22 ± 4
72 h	− 10 ± 10	− 11 ± 2	− 11 ± 4
Water intake, ml			
24 h	76 ± 10	77 ± 12	80 ±14

Values are reported as mean ± SEM

### Plasmatic xanthine oxidase activity

**Fig. 2:**
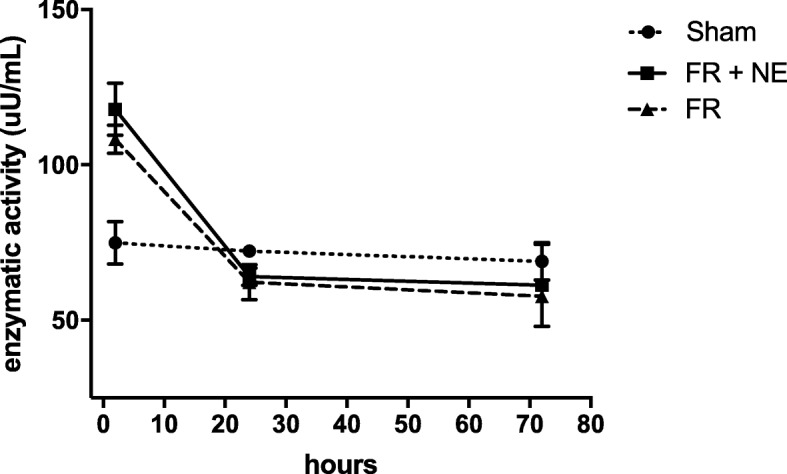
Plasmatic xanthine oxidase. Xanthine oxidase concentration assessed by ELISA after 2, 24 and 72 h of reperfusion and expressed in uU/ml. *N* = 4–5/group at each time point, and asterisk indicates a significant difference between HS vs sham group, (*p* < 0.05)

Xanthine oxidase activity increased after HS, at the end of reperfusion compared to sham (*p* < 0.05); no difference was noted between the resuscitation strategies and xanthine oxidase activity in HS groups decreased to values similar to sham at 24 h and 72 h (Fig. 2).

### Makers of apoptosis: caspase 3

**Fig. 3:**
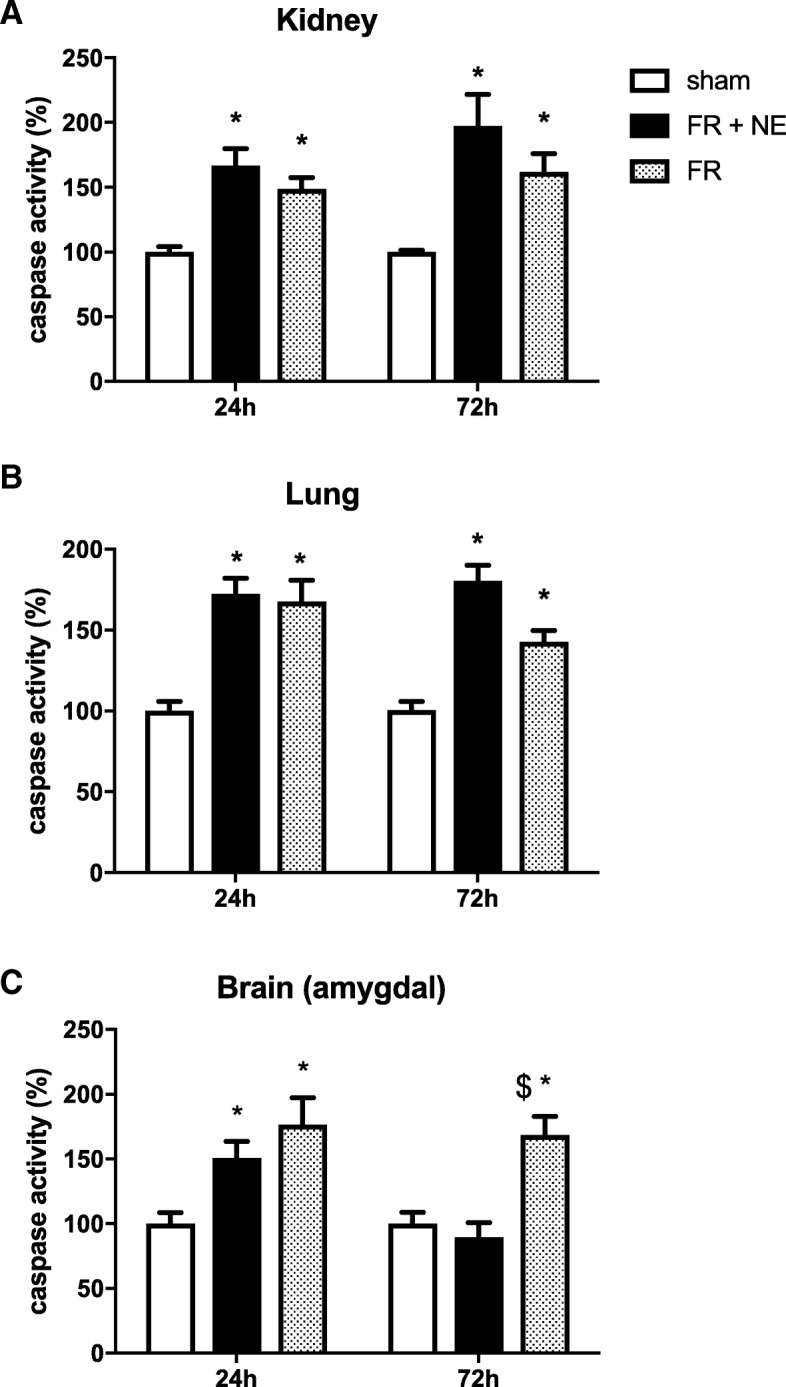
Caspase 3 activity in organs. Caspase 3 activity kidney (**a**), lung (**b**) and brain amygdala (**c**), expressed as the percentage of the mean activity observed in the sham group, set to 100% and assessed by in vitro spectrofluorescence after 24 h and 72 h of reperfusion. *N* = 5/group and asterisk indicates significant difference between both HS groups vs sham group, except for FR+NE group in brain amygdala at 72 h (*p* < 0.05); dollar sign indicates significant difference between FR vs FR+NE in brain amygdala at 72 h (*p* = 0.002)

Caspase 3 activity was measured in the three organs (kidney, Fig 3a; lung, Fig. 3b; brain, Fig. 3c). In the kidney and in the lung, caspase 3 activity increased at 24 h and 72 h following HS compared to sham (*p* < 0.05). No statistically significant difference was present, despite a trend towards higher expression at 72 h in both organs for the FR+NE group. In the brain-amygdala, caspase 3 activity increased at 24 h following both resuscitated HS compared to sham (*p* < 0.05) but was significantly higher in the FR compared to FR+NE at 72 h (*p* = 0.002).

### Neutrophil’s infiltration (MPO in tissues)

**Fig. 4:**
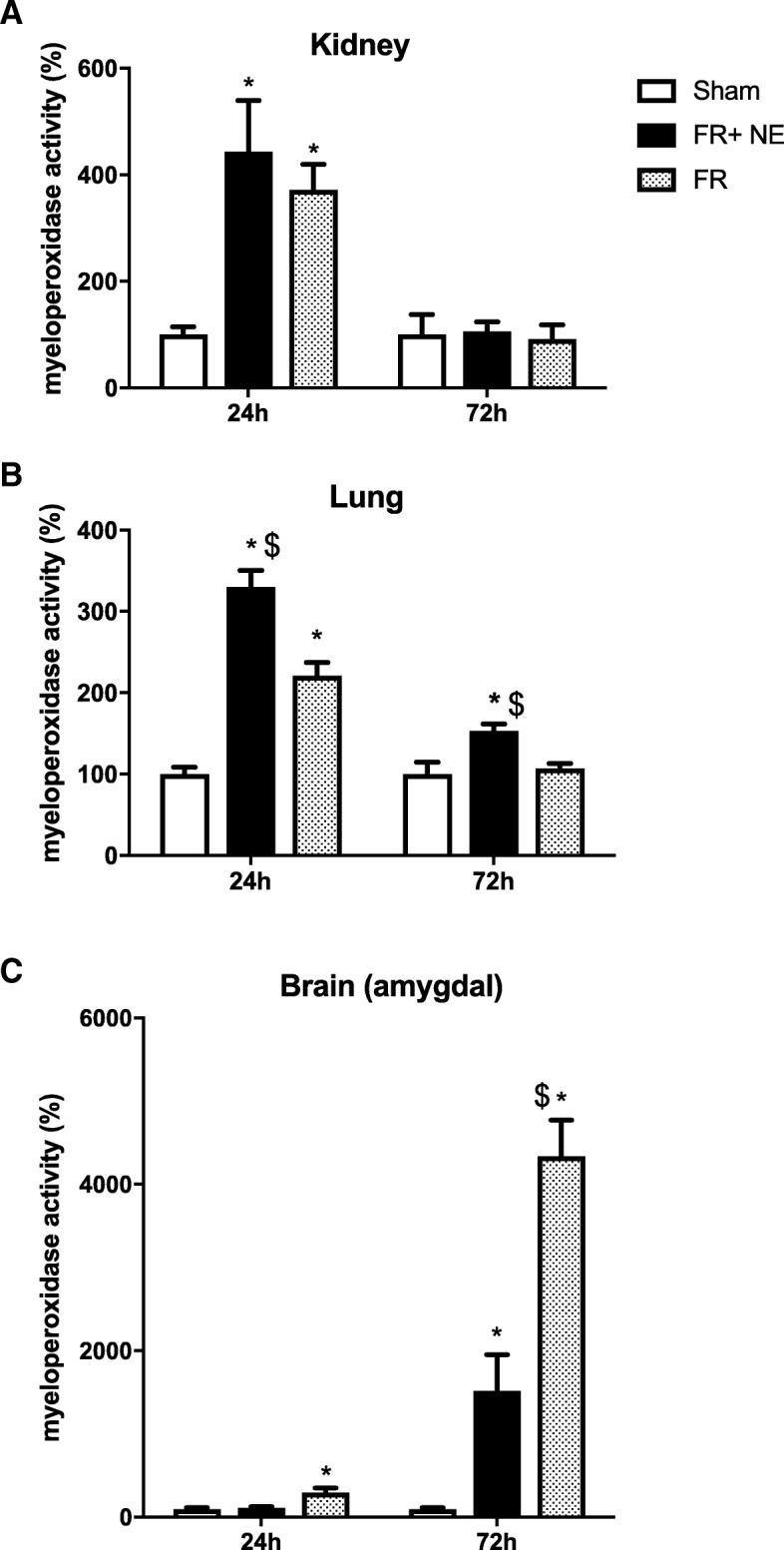
Myeloperoxidase activity in organs. Myeloperoxidase activity in the kidney (**a**), lung (**b**) and brain amygdala (**c**), expressed as percentage of the mean of the sham group set as 100%, assessed by enzymatic reactions after 24 h and 72 h of reperfusion. *N* = 5/group and asterisk indicates significant difference between HS vs sham group and dollar sign indicates FR vs FR+NE (*p* < 0.05)

Myeloperoxidase activity (MPO), representing a surrogate of neutrophils influx, was measured (kidney, Fig 4a; lung, Fig. 4b; brain, Fig. 4c). MPO in the kidney was significantly higher in both HS groups at 24 h of resuscitation (vs sham; *p* < 0.05) but disappeared at 72 h. In the lung, however, HS led to increased MPO which was significantly higher in the FR+NE group compared to FR at 24 h (*p* = 0.001) and 72 h (*p* = 0.007). In the brain amygdala, the HS shock led to increased MPO only after FR at 24 h (vs sham; *p* < 0.05); at 72 h, MPO activity was increased after both HS, but significantly higher after FR compared to FR+NE (*p* < 0.01).

### Protein markers of organ injury after ischemia

**Fig. 5:**
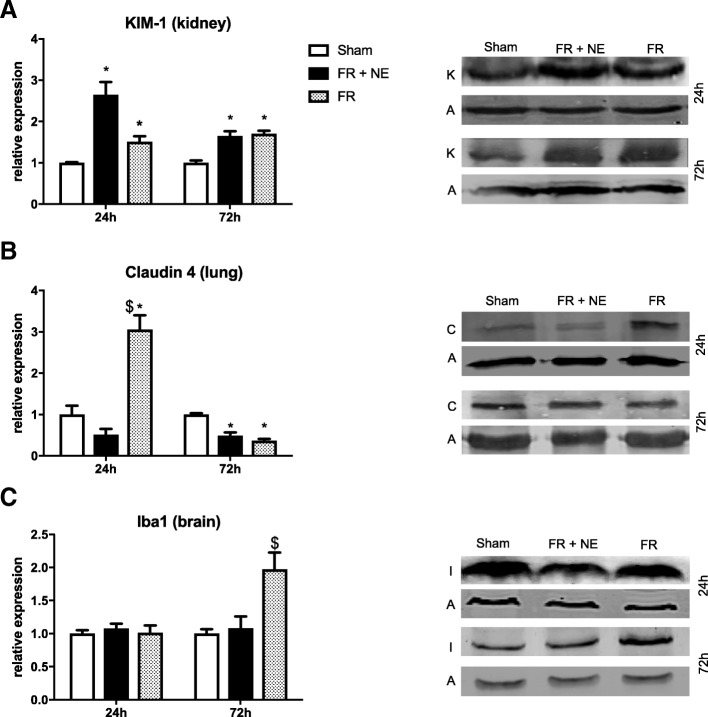
Protein marker of injury in organs. **a** KIM-1/actin ratio (fold changes) expressed in the kidney, assessed by Western blotting in vitro after 24 h and 72 h of reperfusion. **b** Claudin-4/actin ratio expressed in the lung. **c** Iba1/actin ratio expressed in the amygdala of the brain. *N* = 5 and dollar sign indicates significant difference between FR and FR+NE vs sham groups (*p* < 0.05). Side panels are representative Western blots; K: KIM-1, C: Claudin-4, I: Iba1, A: actin

Post HS, the expression of selected proteins was measured in the end organs. In the kidney (Fig. 5a), KIM-1 was increased as expected after HS (*p* < 0.05), but no difference between the resuscitation strategies was visible at both time points, even if it was not significantly higher at 24 h with NE. In the lung (Fig. 5b), Claudin-4 expression was strongly induced after HS in the FR group compared to shams at 24 h (*p* = 0.001), when it decreased slightly with FR+NE. The difference between FR and FR+NE was very significant (*p* < 0.001). Its expression was however reduced at 72 h in both resuscitation strategies, compared to sham (*p* < 0.05). To note that after HS, the lung wet/dry ratio was increased (1.8-fold), without differences between the resuscitation groups (data not shown). In the brain amygdala, no induction of Iba1 was noted after HS (Fig. 5c). After 72 h, Iba1 expression was significantly enhanced in the amygdala after HS only in the FR group vs sham (*p* = 0.035).

## Discussion

Our animal model represents a resuscitation strategy involving the use of a vasopressor once bleeding is controlled and partial volemic resuscitation is achieved. Our results are consistent with previous reports that demonstrate the fluid-sparing effect of vasopressors [7]; indeed, the use of vasopressors allowed us to achieve resuscitation with a volume equal to the haemorrhage amount. This is likely due to venous volume recruitment, increased heart contractility and maintenance of end-organ blood flow [27]. Already in the 1970s, Drees et al. had shown venoconstriction to be able to mobilize blood volume [28]. More recently, animal studies as well as human have demonstrated the effect of norepinephrine on venous return, then improving cardiac output [29–31].

The excess amount of fluid necessary to maintain adequate blood pressure without vasopressors, explained by the probable ischemia-reperfusion induced vasoplegia, confirmed the findings of Dalibon et al. [32]. This vasoplegia hypothesis is also supported by the initial restoration of MAP with smaller volume of blood reinfusion, followed by a need for more fluids to maintain the same MAP target [32]. We could not measure vascular resistance in our model, but we measured xanthine oxidase activity, as one marker of HS-induced vasoplegia [33]. Our observation of increased xanthine oxidase activity after HS was in accordance with what had been described in the past [15]. Neither the higher fluid volumes nor the use of vasopressors modified its expression during or after the resuscitation phase.

Vasopressor therapy did not modify lactate levels, suggesting that restrictive fluid therapy did not increase anaerobic metabolism; it is then unlikely that excessive vasoconstriction was present, in contrast to other model of progressive bleeding [34]. In addition, we found no difference in saturation, PaO_2_, pH and temperature.

In the kidney, despite the peak of creatinine during reperfusion and a slightly higher KIM-1 with vasopressors and less fluid administered, the evolution was identical between the two resuscitation strategies. After HS and reperfusion, the increase of creatinine was in the magnitude (2-fold) of similar models [24] and the higher peak may be explained by the pre-renal effect of fluid sparing in the group with vasopressors. The fact that the ischemic-sensitive expression of kidney injury molecule-1(KIM-1) [20] was temporarily enhanced, even if not significantly, cannot exclude a vasopressor-induced vasoconstriction in a state of relative hypovolemia [35]. The return to baseline levels in each group (Delta creatinine B) was observed in our comparative model, but does not necessarily demonstrate an equivalent recovery of kidney function, given the limitation of sole creatinine measurement [36].

In the lung, the use of vasopressors seemed to enhance neutrophil infiltration until 72 h. It is known that the catecholamine released during HS can modulate the systemic inflammation; particularly, the alpha-adrenergic effect can activate lung neutrophils [37]. On the other end, the chosen lung protein marker Claudin-4 expression was increased at 24 h following HS, after fluid resuscitation only. It is particularly interesting since this junction protein can be highly expressed after lung injury and is involved in the maintenance of alveolar barrier function and fluid clearance [21, 38]; at the later time point, its expression was decreased in both groups, likely secondary to inflammation [39]. Regarding the differential expression at 24 h, we can hypothesize that the fluid volume overload due to resuscitation after ischemia-reperfusion might have triggered a temporary compensatory reaction to enhance fluid clearance; in the group exposed to vasopressor, the observed inhibition might have happened secondary to TNF-α in the context of enhanced inflammation [39]. Further elucidation of the mechanistic reason is needed.

The major differential effect of the two resuscitation strategies, as well as the more congruent findings, was in the brain. To note that in our model, the amygdala was used as a sensitive zone of the brain but does not represent the consequences that might occur in other parts. Fluid resuscitation alone did provoke more caspase 3 activation, more MPO and more microglia activation (Iba1) particularly at the later time point. No difference in brain perfusion pressure was suspected, given the similar MAP during shock or resuscitation. One explanation could be that the excess of fluid administered impacts on blood-brain barrier and contributes to worsen the ischemic amygdala [40]. Early works had already measured the increase of brain damage if resuscitated with fluid after brain ischemia [41]; it has to be verified with more sophisticated measure of permeability. Nevertheless, it supports the beneficial effect of vasopressors use as a fluid sparing, beyond the sole purpose of maintaining MAP for perfusion pressure.

Some authors reported that global ischemia-reperfusion does not increase MPO expression in rat brain [42], when others did [43]. We found a significant increase of MPO after HS at the later time point, particularly if resuscitated with fluid only. The magnitude of our global ischemia and the timing might have allowed the deterioration of blood-brain barrier integrity to develop [44]. These findings parallel the improvement of brain oxygenation and decreased intra-cranial pressure observed after resuscitation using vasopressors instead of fluids only [45].

We acknowledge some limitations of our work. First, our focused approach is limited by the three chosen organs. In addition, our model differs from previous reports, since the frame of our research question targets the post-emergency management period, where the bleeding and its sources have been controlled. It is in contrast with the existing experimental literature, where vasopressors are administered simultaneously during fluid resuscitation [16, 45]. Finally, the biomarkers chosen are only illustrative of specific phenomenon, without relevance for common mechanisms or measurable effect on organ’s function. The selection of them is a matter of debate, and our choices were made among numerous existing biomarker. Additional microcirculatory, as well as more functional assessment of organ integrity/ischemia, could have enhanced the clinical relevance of our findings.

Nevertheless, our observations contribute to support the resuscitation approach using vasopressors as fluid sparing once the haemorrhage is controlled, particularly for the brain. It is also reassuring regarding the impact on the kidney, particularly given the known recognized association between fluid overload and acute kidney injury. As for the lung, the limitation of fluids administration is already an accepted principle among clinicians, particularly after lung transplantation and ARDS.

## Conclusion

Our data show that the organ perfusion can be assured with lesser fluid administration followed by vasopressors administration in order to resuscitate HS, once controlled. No sign of dysoxia or worst evolution is provoked with this approach. Even more, it suggests that limiting the fluid administration could benefit the brain and seems not to have a negative impact on the lung or kidney.

## Abbreviations

FR+NE: Fluid resuscitation and norepinephrine
FR: Fluid resuscitation
HR: Heart rate
HS: Haemorrhage shock
Iba1: Ionized calcium binding adaptor molecule 1
KIM-1: Kidney injury molecule-1
MAP: Mean arterial pressure
MPO: Myeloperoxidase activity
